# Variability in Regularity: Mining Temporal Mobility Patterns in London, Singapore and Beijing Using Smart-Card Data

**DOI:** 10.1371/journal.pone.0149222

**Published:** 2016-02-12

**Authors:** Chen Zhong, Michael Batty, Ed Manley, Jiaqiu Wang, Zijia Wang, Feng Chen, Gerhard Schmitt

**Affiliations:** 1 Centre for Advanced Spatial Analysis, University College London, London, United Kingdom; 2 School of Civil and Architectural Engineering, Beijing Jiaotong University, No.3 Shangyuancun, Haidian District, Beijing, P. R. China; 3 Beijing Engineering and Technology Research Centre of Rail Transit Line Safety and Disaster Prevention, No.3 Shangyuancun, Haidian District, Beijing, P. R. China; 4 Future Cities Laboratory, Department of Architecture, ETH Zurich, Zurich, Switzerland; University of Warwick, UNITED KINGDOM

## Abstract

To discover regularities in human mobility is of fundamental importance to our understanding of urban dynamics, and essential to city and transport planning, urban management and policymaking. Previous research has revealed universal regularities at mainly aggregated spatio-temporal scales but when we zoom into finer scales, considerable heterogeneity and diversity is observed instead. The fundamental question we address in this paper is at what scales are the regularities we detect stable, explicable, and sustainable. This paper thus proposes a basic measure of variability to assess the stability of such regularities focusing mainly on changes over a range of temporal scales. We demonstrate this by comparing regularities in the urban mobility patterns in three world cities, namely London, Singapore and Beijing using one-week of smart-card data. The results show that variations in regularity scale as non-linear functions of the temporal resolution, which we measure over a scale from 1 minute to 24 hours thus reflecting the diurnal cycle of human mobility. A particularly dramatic increase in variability occurs up to the temporal scale of about 15 minutes in all three cities and this implies that limits exist when we look forward or backward with respect to making short-term predictions. The degree of regularity varies in fact from city to city with Beijing and Singapore showing higher regularity in comparison to London across all temporal scales. A detailed discussion is provided, which relates the analysis to various characteristics of the three cities. In summary, this work contributes to a deeper understanding of regularities in patterns of transit use from variations in volumes of travellers entering subway stations, it establishes a generic analytical framework for comparative studies using urban mobility data, and it provides key points for the management of variability by policy-makers intent on for making the travel experience more amenable.

## Introduction

Urban mobility shapes space as much as space shapes urban mobility [1]. To find regularity in human mobility is of fundamental importance to a better understanding of urban dynamics and this yields insights into extensive applications varying from urban transportation [2–4], social structure [5], and urban design [6–8] to epidemiology [9, 10] and urban infrastructure [11]. Urban dynamics can be characterised by mobility patterns at different scales. In terms of the temporal dimension, allometric scaling laws for city size have been discovered from long-term population data [11–13], while patterns of spatial interaction have been explored and modelled over long-time periods and for long-distance movements between cities [14] using power laws.

Urban mobility data has exploded in recent years as data sets pertaining to transactions and movement in real time from mobile phones, GPS tracking, Wi-Fi, smart cards, and social media give much finer granularity of detail. This has greatly promoted the discovery of many different kinds of regularities, adding new perspectives to classical scaling laws and theories, especially for short-term movements at an individual level. For instance, Gonzalez et al [15] tracked anonymised mobile phone users for six-months, finding, in contrast to the random trajectories predicted by the prevailing Levy flight and random walk models, a high degree of spatiotemporal regularity exists in human trajectories. Schneider et al [16] constructed networks of individual daily mobility from two types of data, namely mobile phone data in Paris and trip survey data in Paris and Chicago, finding 17 unique motifs that all follow simple rules useful for modelling and simulation. Other work using multi-source data including taxi data has suggested that as population density decreases exponentially with distance from the urban centre, this ultimately leading to an exponential law of collective intra-city mobility [17].

There are also studies using smart card data, a comparatively new type of data generated by Smart Card Automatic Fare Collection (SCAFC) systems. These data have revealed diverse features about mobility that have not been possible to observe hitherto. The small world phenomenon, for example, has been found in daily encounters relating to shared bus travel establishing certain probabilities of meeting “familiar strangers” [18]. A similar phenomenon has been found in the geographic circulation of banknotes in the United States [19]. Other data sets form social media sites such as Foursquare [20] differ from data where mobility is directly deterred by the costs associated with physical distance, generating scaling laws that are consistent with intervening opportunities, built on rank-distance instead of pure physical distance.

Though progress has been made in revealing different perspectives on regularity as well as adding variability at finer scales to classical universal scaling laws, the statistical structure of human mobility is still far from predictable. High degrees of regularity emerge mostly at aggregated levels either for large population groups or for long-term changes. Detected preferences for movements at fine scales against more simplistic laws of motion already demonstrate the existence of such variability and this has been briefly addressed in recent work [15, 20, 21]. At a disaggregate level, diversity appears due to increasingly complex causal factors. Song et al [22] raise a fundamental question as to what degree is human behaviour predictable from investigations of the stability of predictions by measuring the entropy of individual trajectories. Factors such as travel time, sample size, as well as demographic structure are discussed to explain these entropy values. In the light of the insights gained in this research, we hypothesize that in the context of urban mobility, the degree of regularity decreases across all scales, specifically, spatial and temporal scales, and aggregations of different factors that condition mobility itself, and thus follow certain functions that can be detected, represented and then modelled.

Here we raise three questions. First at which aggregated scales does regularity persist? Second if certain changes in regularity occur, does this occur randomly or does it scale in a regular way? Third, does urban context matter? This paper investigates such ‘variability in regularity’ with a primary focus on temporal scales. The reason we begin with the temporal dimension is to make use of the distinctive advantages of smart-card data, which enable us to look into urban mobility down to the scale of one-minute granularity, which is as detailed as any analysis of such urban phenomena to date.

We first need to clarify definitions. By *regularity*, we mean a uniform pattern, principle, arrangement, or order that repeats itself, is reproducible, and therefore can be used as basis for urban and transport simulation and prediction. By *temporal scale*, we mean the minimum temporal unit for data aggregation, which implies how far one is able to look at the temporal series backwards and forwards. We will propose a method to quantify the stability of any form of regularity identified by measuring variability over different temporal scales. The method is applied to smart-card data, which has wide demographic and geographic coverage of urban mobility. We use this variability in regularity as an index for comparative study using one-week’s worth of smart-card data from our three candidate cities, namely London, Singapore and Beijing. On the one hand, our purpose is to demonstrate the existence of universal regularity as variability changes; on the other, our approach serves as a common analytical framework for comparing regularities in human mobility, relating any variability to related characteristics of cities.

## Methods: A Basic Measure of Regularity

We give scope to this work by evaluating the stability of regularity in the temporal dimension through measuring the variability of mobility patterns over multiple days. We assume here that the units associated with regularity could be any elements or objects that are associated with urban mobility, such as individual passengers, trains, stations, flows and so on. Accordingly, a set of attributes will be defined to characterize a specific regularity; for instance, travel purpose for a person, capacity and speed for a train, transfer flows for a station, and so on. Although our comparative studies in later sections use stations as study subjects specifically, we will also generalise our method to flow systems that depend on trips between origins (*O*) and destinations (*D*). The method we propose is thus generic and applicable to many conceptualisations and aggregations of our basic data sets.

### 2.1 Definitions and Notation

Urban mobility can be decomposed and evaluated in terms of different objects, such as a traveller, a station, an area associated with a station, or a fixed transit line or route. An object with finite measure *x*_*t*_ can be written as a vector **x**_*N*_(*i*)

$$x_{N}(i)=[x_{1},x_{2},x_{3},\ldots \;x_{t},\ldots \;x_{n}]$$ (1)

where *x*_*t*_ is a measure of the object *N*, say a station, at time slot *t*, *t* ∈ [1, …, *n*] and *n* is the number of sequenced time slots; [*x*_1_,*x*_2_,*x*_3_,… *x*_*t*_,… *x*_*n*_] is thus the temporal pattern of the object **x**_*N*_(*i*) which depends on the number of time slots *n* that define a day. Moreover, *i* denotes the day on which the temporal pattern is measured. A stable and reliable regularity would thus exist with respect to this profile over days implying that **x**_*N*_(*i*) ∼ **x**_*N*_(*j*), *i* ≠ *j*. The objective of our analysis is to assess the variability of **x**_*N*_(*i*) between multiple days. Note that the index *N* characterises the object in question that might be a set of stations or a set of travellers and it is clear enough that we do not need to notate each element of this vector *x*_*t*_ with respect to its object *N* or day *i*.

Moreover, an individual measure *x*_*t*_ could be a single value, for instance, the number of people who enter a station at time *t*. *x*_*t*_ itself could also be a vector if there is more than one attribute used describe the pattern. For instance, the number of people holding different types of smart card could further decompose the number of passengers into *m* attributes and in this way we might define a matrix **X**_*N*_(*i*) as

$$X_{N}(i)=\left[\begin{array}{l} x_{11},x_{12},x_{13},\ldots \;x_{1t},\ldots \;x_{1n}\\ x_{21},x_{22},x_{23},\ldots \;x_{2t},\ldots \;x_{2n}\\ x_{31},x_{32},x_{33},\ldots \;x_{3t},\ldots \;x_{3n}\\ \;.\;.\;.\\ x_{m1},x_{m2},x_{m3},\ldots \;x_{mt},\ldots \;x_{mn} \end{array}\right]$$ (2)

where each column is the vector associated with *x*_*t*_ in Eq (1) above. To repeat the limits, *N* is the object type, *i* is the day, *n* is the number of sequenced time slots, and *m* is the attribute of the element defined at the time slot in question. In the sequel, we assume that the time slots are in minute intervals, the attributes are the types of travel such as the flow from one station to another, the objects are defined with respect to the station they profile, and the day of the week for this profile defines the station and the elements of the matrix **X**_*N*_(*i*). We should also note that Eq (2) is a generalisation of Eq (1) in that if, then the problem collapses to one where we are examining variations across attributes of passengers. When *m* = 1, the variation is across temporal intervals.

### 2.2 Measuring Accumulated Variability

We first consider the vector **x**_*N*_(*i*) which we will use to measure the relative variance of each distribution over multiple days. Since there is no baseline for comparison, we compute the variability between any two distributions for each of two separate days. Regularity exists only when the variance between any two distributions is low, and we will reject the hypothesis that there is strong regularity if the detected temporal patterns across certain time units are unstable over multiple days. We measure this degree of regularity from the correlation between any two days *i* and *j*, *i* ≠ *j* where we generate the correlation between **x**_*N*_(*i*) and **x**_*N*_(*j*) from the normalised covariance matrix formed from these vectors. This gives the correlation matrix *r*(*i*, *j*) from which we can gauge the degree of regularity between any two days.

The square of these values *r*^2^(*i*, *j*) is the amount of covariance that is explained by the correlation where, for example, if this is 0.6, then this means that 60% of the variation between any two patterns or profiles **x**_*N*_(*i*) and **x**_*N*_(*j*) is common and the remaining 40% is not. Clearly in this context, we are looking for comparisons where the *r*^2^(*i*, *j*) values are as high as possible. Since variance between any two days is what matters, we can calculate the accumulated variance, which we define as the variability statistic as:

$$CVar=\sum_{i=1}^{l}\sum_{j=i+1}^{l}\left(1-r^{2}(i,j)\right)/\left(l(l-1)/2)\right)$$ (3)

where the summations are over *l* days, normalized by (*l*(*l*−1)/2)) which is the number of comparisons.

As we aggregate the time interval during a day composed of 1440 minutes (which is the temporal resolution of the smart card data that we have), we expect that the variability of the profile will decrease and that a more regular profile will exist in terms of the comparison between days. In short, if we fix the time interval as *T* minutes, then we have *n* = 1440/*T* intervals defining the profile **x**_*N*_(*i*). When we reduce this interval, the associated temporal patterns of flows show greater variability due to the fact that detailed decisions as to arrival times incorporate greater flexibility. Actually, in any data set that is aggregated from individual observations, the variability of individual observations decreases due to averaging of the data. Given this determinant of variability, we are searching for deviations from this pattern between days and between cities and dependent on causal determinants related to when people travel, then the variability measure *CVar* in Eq (3) will detect such differences. The deviations/variability determines to what extend history trip data can be used for real-time trip prediction, which is crucial for passenger management, and transit planning.

## Data and Applications

Our comparative study of temporal urban mobility patterns using the proposed variability has been developed for three large cities, namely London, Singapore, Beijing, all of which have available smart-card data for at least one week. The smart-card data is generated by various Smart Card Automated Fare Collection (SCAFC) systems, which were originally designed to collect revenue for better financial management and accounting for public transportation systems. These also produce large volumes of data about boarding and alighting from the vehicles or trains which in the case of the smart-card involve tap-ins and tap-outs by the traveller [23–25].

The quality of smart-card data varies from city to city in term of the richness of the information available, the coding system adopted, and the granularity and completeness of data, but in this analysis, we have only used the basic information including station code and time tag when boarding or alighting occurs. This basic information is captured in almost all the cities for which smart-card data is available, and this implies that this comparative study can be easily expanded.

We will note the key characteristics of the three cities and their transit systems before we begin the analysis. London’s population, which is directly served by the subway and related transit systems, was about 8.6 million in 2014 and its metro system is the oldest rapid transit system in any city, and being first constructed as an underground heavy rail system in the 1860s. The London Travel Demand Survey (LTDS) reports that around 30% of total population in the Greater London area use public transportation for their daily commuting [26]. The smart card data is recorded by London’s Oyster card system which is used by approximately 90% of bus passengers and 80% of rail passengers [27]. The trains dataset contains approximately 9 million transactions daily, which are entry/exit transactions associated with the train systems (Underground (Tube), Overground and Docklands Light Railway (DLR)). Both entry and exit data are available for train rides at gated stations, while only entry data is available for bus rides. During the period of analysis, the London Underground network had 13 lines with 400 stations in use detected from the smart-card data available for a week in February 2014.

Singapore, our second example is an island city-state with a current population of approximately 5.3 million of whom about 62% (or 3.29 million) are residents, the rest being foreign workers or their dependents, according to the 2012 census. The metro system called the Mass Rapid Transit (MRT) system in Singapore, has 102 subway stations with several new lines opening in the last five years. At present the land-based public transportation system in Singapore comprises two networks: the MRT and the bus system with more than half the population using public transportation as their main transport mode [28]. The collected tap-in/tap-out events provide a huge data set with around 5 million daily travel records, which we have been able to access as smart card data provided by the Singapore Land Transport Authority. This study was conducted using the available smart-card data from the EZ-Link system for one week in March 2013.

As the first metro system in mainland China, Beijing Metro system came into service in 1969. About 23.6 km of metro line with 17 stations was built, which was based on military considerations and was open for citizens years later. It has undergone rapid expansion since the end of the 29th summer Olympic Games in 2001. New lines begin operating almost every year. The trip share of metro soar from 3.6% in 2000 to 20.6% in 2013, and is expected to continue to increase. Beijing’s population had reached 21 million by the end of 2013 but a considerable part of the city is not developed being rural and mountainous areas (Beijing Traffic Development Research Centre, 2014). The service area of the metro network in fact is circled by the 6th ring road, and this reduces the population served by the system to some 5 million. The mode share of the metro is 21% without walking trips and the data used for this analysis is based on data from the Yikatong card which accounts for 85% of the total trips made on the network. Although a simple flat fare is applied to the network, passengers need to use their cards to tap in and tap out with two records being logged for each metro trip. The original card transaction data generated on the Beijing metro network were made available for October 2014 by the Beijing Municipal Commission of Transport.

The differences reflected in Table 1 are considerable and before the analysis, we had little idea of the degree to which travel behaviour on these respective subway systems would differ. London’s much older system where there is less scope for new lines than in Singapore contrasts with the Beijing system, which is quite old but has received considerable enhancement in recent years. This is reflected to an extent in usage with the highest usage rate in terms of ridership and mode share in Singapore (35% according to [29]).

**Table 1:** Summary statistics of one-week of smart-card data (metro trips only).

	London	Singapore	Beijing
**Monday**	3,457,234	2,208,173	4,577,500
**Tuesday**	3,621,983	2,250,597	4,421,737
**Wednesday**	3,677,807	2,277,850	4,564,335
**Thursday**	3,667,126	2,276,408	4,582,144
**Friday**	3,762,336	2,409,600	4,880,267
**Number of stations** (1)	400	130	233
**Number of tube line**	13	4	17
**Area** (2)	1,572 km^2^	718.3 km^2^	2267 km^2^
**Total population** (3)	8.63 million	5.3 million	21.15 million
**Ridership of Metro**	20%	35%	21%
**Length of metro lines**	402km	182km (MRT+LRT)	465 km

(1) Number of stations is the number of stations with smart-card records generated.

(2) The area of Beijing only counts the area enclosed by the 6th ring road for a fair comparison.

(3) From the World Population Review, http://worldpopulationreview.com/world-cities/ accessed 05 February 2016

## Experiments and Outcomes

In our experiments, we take the metro station as the basic urban element or object and study the regularity of temporal urban mobility patterns at each station during one-week (5 week days) based on the availability of data in all three cities. Weekends are excluded since huge variability exists on Saturdays and Sundays due to the fact that work and leisure activities differ most during these periods due to cultural factors [30].

We have developed two experiments demonstrating the usage of vector **x**_*N*_(*i*) and matrix **X**_*N*_(*i*) respectively to measure the regularity at different scales with respect to when and where people travel. In our first experiments, we examine when people travel. More specifically, this is about the temporal distribution of trip starting times during one week. In this context, the regularity is about temporal patterns of traffic flow, thus, an individual measure *x*_*t*_ is a single value, which is the gate count. Therefore, we can then derive the definition from the generic vector **x**_**N**_(*i*). We define **f**_*N*_(*i*) = [*f*_1_, *f*_2_, *f*_3_,…,*f*_*t*_,…,*f*_*n*_] where *f*_*t*_ is the number of people starting a trip at a station *N* during time slot *t*. The total number of time slots *n* is determined on a time interval, which is defined to aggregate the basic minute by minute data. For instance, if the time interval is set to be 12 hours, then *n* = 2 because a day will be divided into two time slots. Note that there are 1440 minutes in a day and the time interval for dividing a day in 2 would be of 720 minutes duration.

The second experiment conflates the data to deal with flows between origins and destinations, which are associated with each station *N*. In this case, an individual measure *x*_*t*_ has more than one attribute, which are interchange flows between any pair of stations. The regularity is therefore based on spatial patterns of trips, which originate and are destined for each station. We then derive the definition from the generic matrix **X**_*N*_(*i*). Each measure *f*_*t*_ is a vector composed by 2(*M* − 1) attributes that are volume of passengers flowing from the given station *N* as an origin to all other stations and from all other stations to the destination station *N* in question for a given day *i*. Following Eq (2), we can write this matrix as

$$F_{N}(i)=\left[\begin{array}{l} f_{N,1},\;f_{N,2},\;f_{N,3},\ldots \;f_{N,t},\ldots \;f_{N,n}\\ f_{N-1,1},\;f_{N-1,2},\;f_{N-1,3},\ldots \;f_{N-1,t},\ldots \;f_{N-1,n}\\ f_{N-2,1},\;f_{N-2,2},\;f_{N-2,3},\ldots \;f_{N-2,t},\ldots \;f_{N-2,n}\\ \;.\;.\;.\;.\;.\\ f_{M,1},\;f_{M,2},\;f_{M,3},\ldots \;f_{M},t,\ldots \;f_{M,n}\\ \;.\;.\;.\;.\;.\\ f_{M,1},\;f_{M,2},\;f_{M,3},\ldots \;f_{M,t},\ldots \;f_{M,n}\\ f_{M-1,1},\;f_{M-1,2},\;f_{M-1,3},\ldots \;f_{M-1,t},\ldots \;f_{M-1,n}\\ f_{M-2,1},\;f_{M-2,2},\;f_{M-2,3},\ldots \;f_{M-2,t},\ldots \;f_{M-2,n}\\ \;.\;.\;.\;.\;.\\ f_{N,1},\;f_{N,2},\;f_{N,3},\ldots \;f_{N,t},\ldots \;f_{N,n} \end{array}\right].$$ (4)

This matrix is arranged so that the columns represent the outflow of passengers from one station *N* to all other stations up to *M* and the inflow from all other stations starting from *M* to the station in question *N* where the range *N*…*M* is the outflow and the range *M* …*N* is the inflow. In short for each time interval, we have a vector of length 2(*M* − 1) where the number of stations is *M* and the self flows from a station to itself are also counted. The first part of the column vector in the matrix is the outflow and the second part the inflow, thus accounting for all flows from and two each station in question. The matrix **F**_*N*_(*i*) are these flows for a particular station and the matrix is thus a measure of the variability over all time periods and over all flows to all stations for a particular station in question. A comparison of matrices **F**_*N*_(*i*) and **F**_*N*_(*j*) for each station provides the variability at this more detailed level of trip flows. There flows represent the trips from an origin station to all its destinations and from these destinations back to the origin station.

What we will do is test the relative stability of the flow profiles for each station across all weekdays, that is assess the extent to which **F**_*N*_(*i*)∼**F**_*N*_(*j*) across all days at different scales. The temporal scales in question cover all aggregations of the day in terms of minutes with *n* varying from 1440 where each interval is one minute to 2 intervals which is the day divided into two equal periods of 12 hours. Thus the number of intervals is always a divisor of 1440 minutes which will give equal time intervals. We do not use all possible intervals as those that we use here are thus defined as intervals of 1440, 720, 360, 96, 80, 60, 45, 30, 20, 15, 8, 4, 2, and 1 minutes which yield *n* = 1,2,4,15,18,24,32,48,72,96,180,360,720,1440 numbers of elements. The rationale for this sequence is to keep the intervals as close as possible to some exponentially increasing function of time over the range from 1 to 1440 minutes.

### 4.1 Variability Increases on Lower Temporal Scales

When and where people travel to is the most fundamental information that can be extracted from urban mobility data and plots of temporal distributions with respect to when a trip starts or ends can be found in most of the results from mobility data analysis [31, 32]. Usually very regular distributions over multiple weekdays can be easily be spotted with very similar flow volumes especially at peak hour times. The O-D matrix is also another frequently used plot for transit planning, which is used in estimating flows between locations. Both temporal distributions and the O-D matrix profiles can be generated at different temporal scales. As indicated earlier, the choice of different scales implies how far one is able to look back and how far forward or ahead. Using this focus on temporal scales, we can interpret the results from our analysis in terms of four related questions based on 1) how does regularity change at each temporal scale? 2) how does regularity change across temporal scales? 3) how does regularity vary within each temporal scale? and 4) how easy is it to predict destination choices of trips at any time of the day? We pose these questions rhetorically and answer them in turn.

#### 4.1.1 Does detected regularity sustain at all temporal scales?

**Fig 1:**
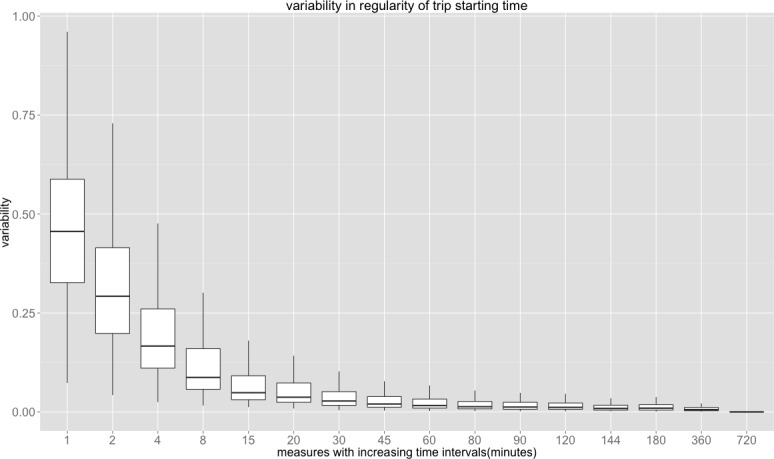
Variability of temporal patterns of trip starting times on the London underground.

**Fig 2:**
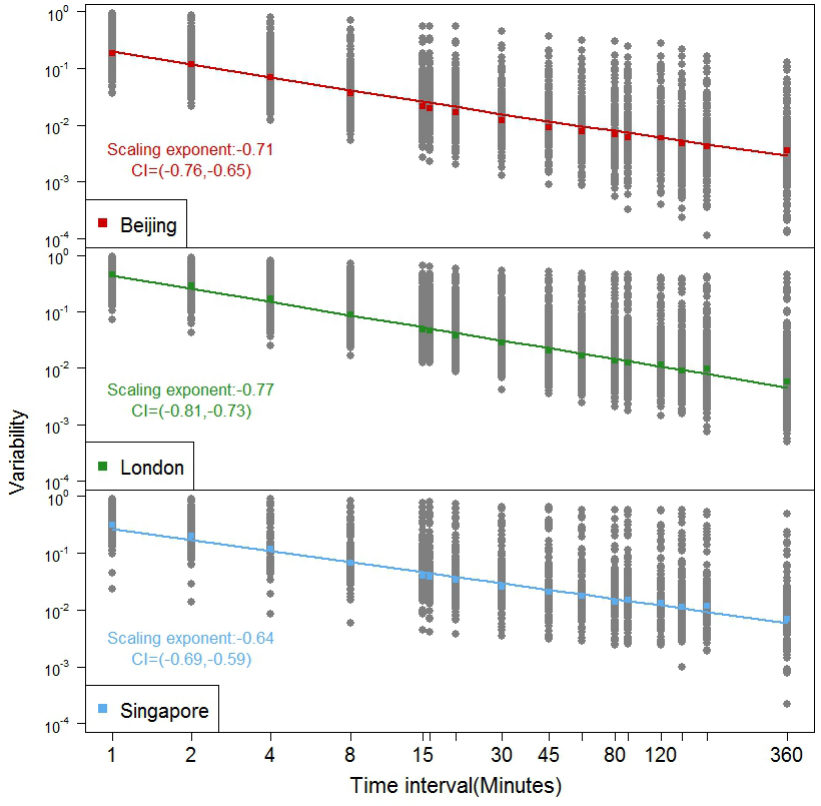
Variability as a function of the time intervals for London, Singapore, and Beijing. Note: The negative linear relations show that the variability declines with increasing temporal scale but the variations in the variability (the variance of the covariance) which relate to the stations also increase for all three cities.

The degree of regularity clearly decreases as the temporal dimension gets larger, that is as the scale gets coarser. Fig 1 is a plot of the normalised variability *CVar* of all measures for all stations using defined time intervals from 1 minute to 12 hours which are displayed left to right on x-axis. A higher variability means less regularity, in other words, the regularity becomes more difficult to predict with accuracy. Fig 2 also shows that the variability increases along with increasing temporal resolution. This is in line with our notion that passengers arrive at stations randomly with respect to their decision to travel and this is a product of human decision, congestion at the gate, bad weather, and human frailties in terms of keeping to their schedules and so on.

#### 4.1.2 How does the detected regularity change across temporal scales?

If we arrange the scales from the smallest to the largest as in Fig 1, the variability decreases in a regular fashion following a non-linear function, which we show in Fig 2 where the vertical scale is logged to assess the degree of linearity. Here we show this for the three cities London, Singapore, and Beijing and this implies that the same kind of exponential decrease in variability occurs that we see in Fig 1 for Singapore and Beijing. London has the steepest scaling exponent of -0.772 whereas Singapore has the lowest scaling -0.645 but the differences do not appear particularly significant. Note that in Fig 2, the median value of *CVar* is plotted as are all other values for each of the metro stations for each city. The median values are then used to fit the linear relations over the 17 time intervals in question and it is clear that the relations are essentially non-linear reflecting the consistency with Fig 1, which just shows the London underground.

#### 4.1.3 At each temporal scale, how does variability in regularity vary?

**Fig 3:**
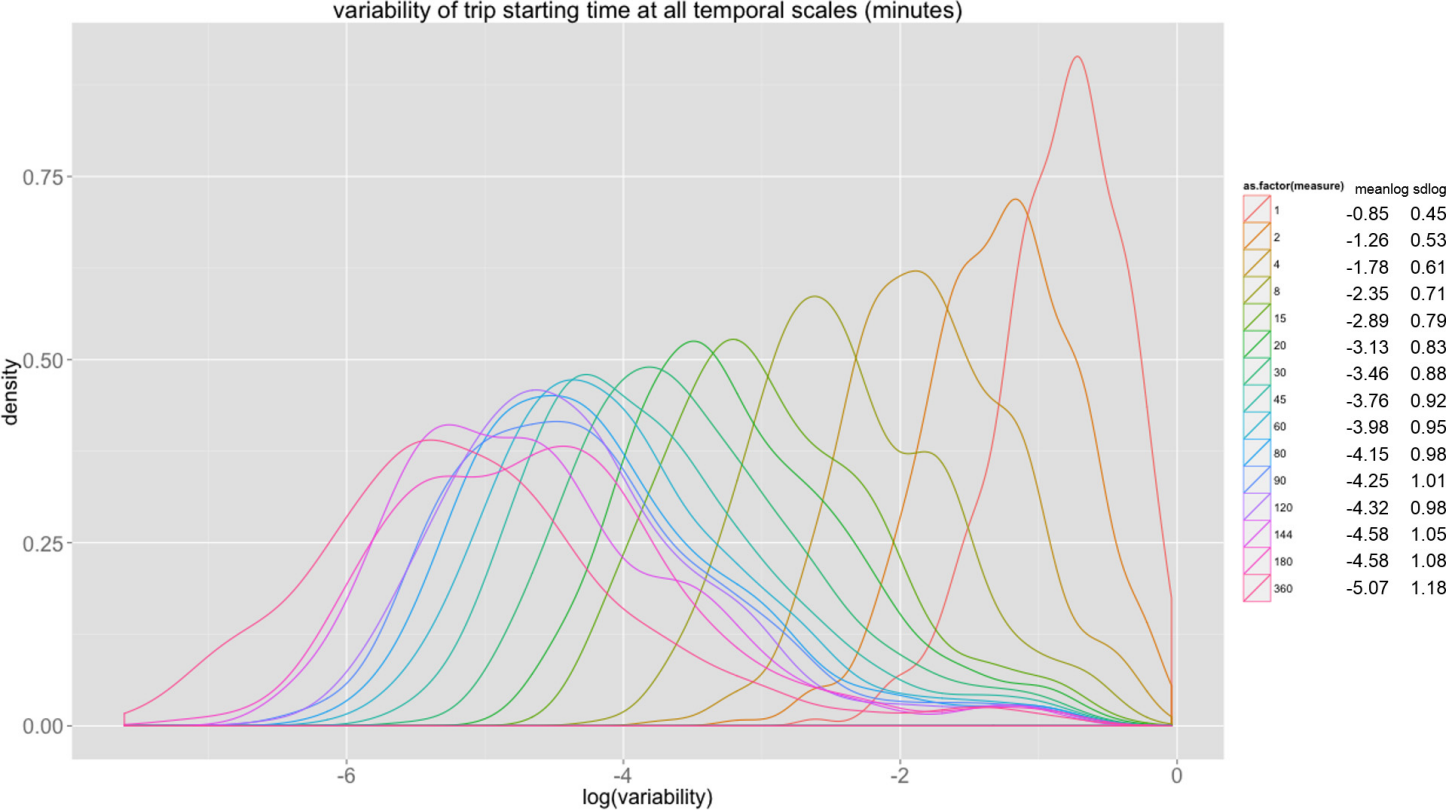
Distribution of the variability of trip starting time at London underground stations. Note: Less variance as the temporal scale gets finer while log normality is consistent over all scales.

Fig 3 plots out the density distributions with respect to the variability of all 400 stations measured at different temporal scales for London. Different colours denote measurements at different time intervals. These density distributions of the variances associated with stations imply less variance as the temporal scale increases and is consistent with Fig 2 while this log normality is consistent over all scales. These results partially prove our hypothesis that regularity can always be found at the aggregated level while diversity appears at the disaggregate.

#### 4.1.4 Can we predict the variability of all trips at all times of a day?

**Fig 4:**
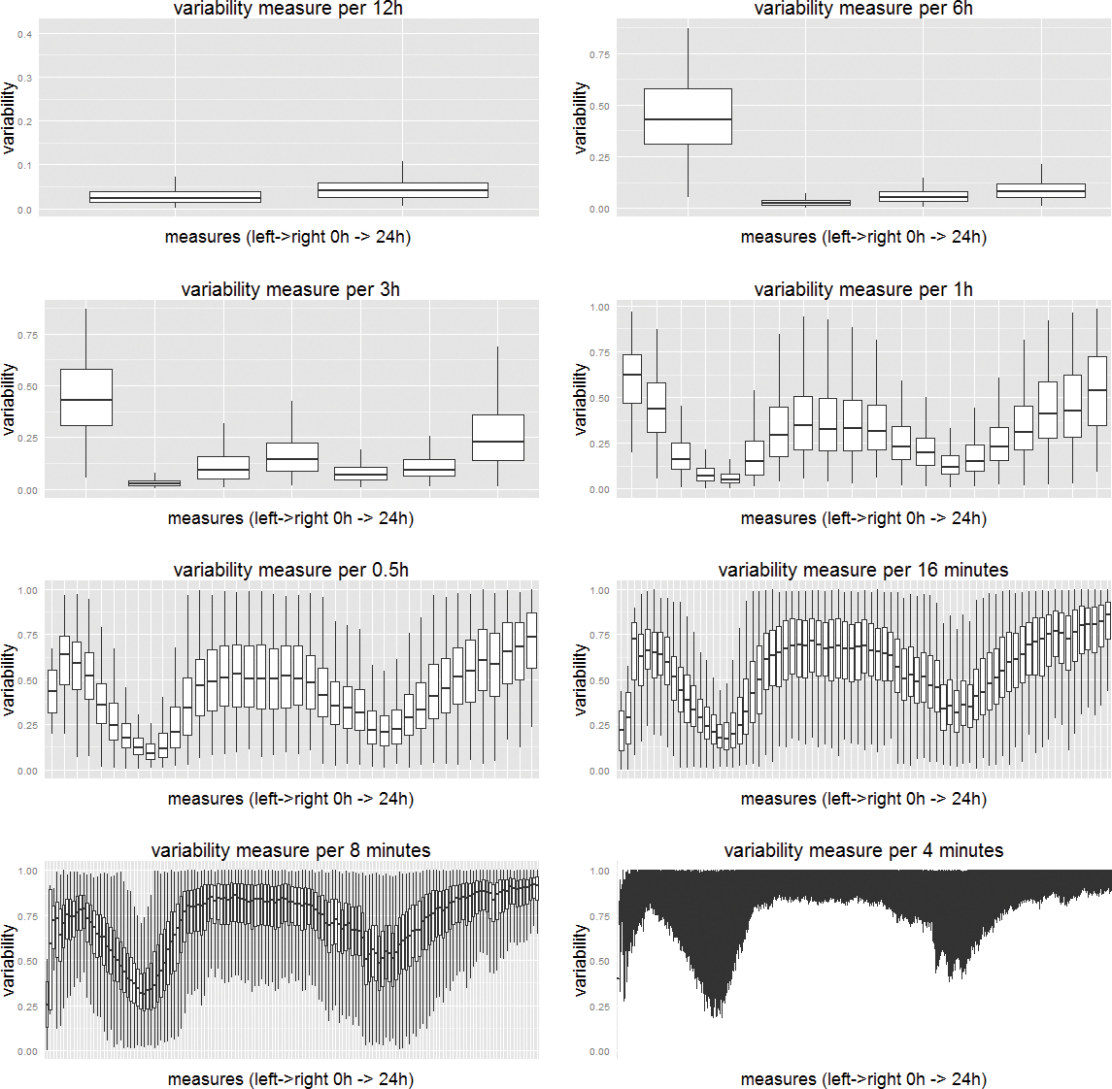
Variability of regularity in the trip matrix over time. Note: Each box plot shows the variability of 400 stations over time measured at different temporal scales. Overall, eight subplots give a similar trend where lower variability appears during peak hours (around 9 am in the morning and 6pm in the evening). More details can be captured as differences of variability between each time unit are magnified as we decrease the temporal scale from 12h to 4 minutes.

This question concerns the variability of trips at different stations at different times of the day. This is important because the data that forms the essence of the Origin-Destination (O-D) matrix for estimating traffic flows is an important problem where smart-card data has already been used implying a fair degree of accuracy [27, 33]. In the same way that we examined changes in overall variability with respect to different temporal scales, the variability of trips at each time of the day clearly gets larger for finer temporal scales and this is clearly shown in Fig 4. Moreover, the variability of trips during different times of the day implies different distributions. Morning and evening peak hours have a comparatively lower variability across all measures. In other words, trips in the peak hours are most regular and thus more predictable probably because most of them are home-work based trips that have fixed origins and destinations. We thus conclude that the distribution of trips can be predicted at aggregated levels but only for specific periods of a day, namely the peaks. To an extent, this demonstrates that focusing transport models on predicting peak hour flows is statistically more robust than using them for prediction at other times of the day.

**Fig 5:**
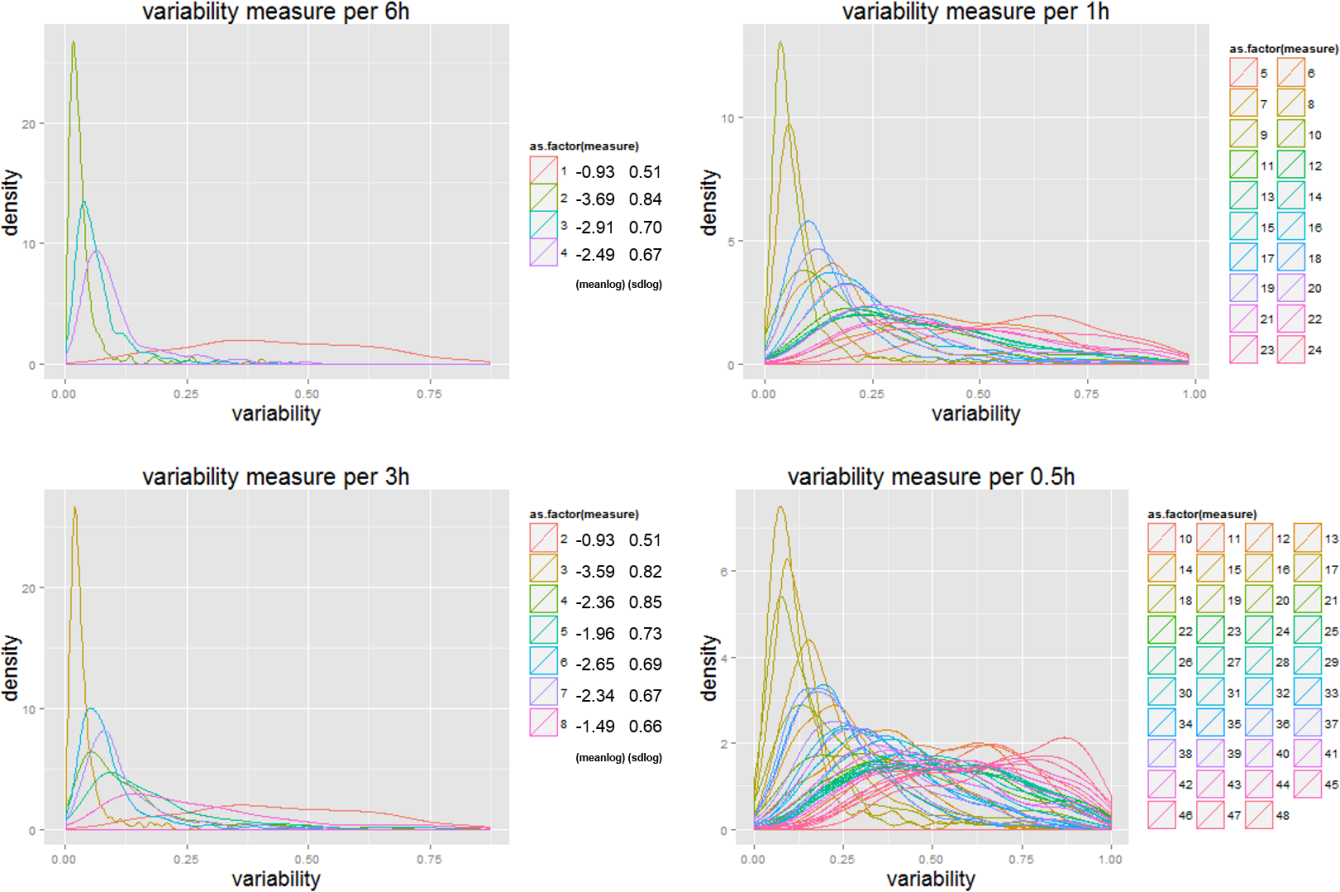
Densities of variability for different time intervals for 400 London underground stations. Note: Less variance exists at a certain time slot, while log normality is consistent over all time slots at all scales. More details about the differences show up when the temporal scale decreases.

As in Fig 3, we also plot out the density distributions of variability for the 400 London underground stations, which we show in Fig 5. The four plots are taken from results for the 6 hour, 3 hour, 1 hour, 0.5 (half) hour time intervals respectively. These distributions for the different time slots are shown in different colours. A lognormal like distribution can be found for all measures, with smallest variance values at the morning and evening peaks.

### 4.2 Regularity Rankings for London, Singapore and Beijing

**Fig 6:**
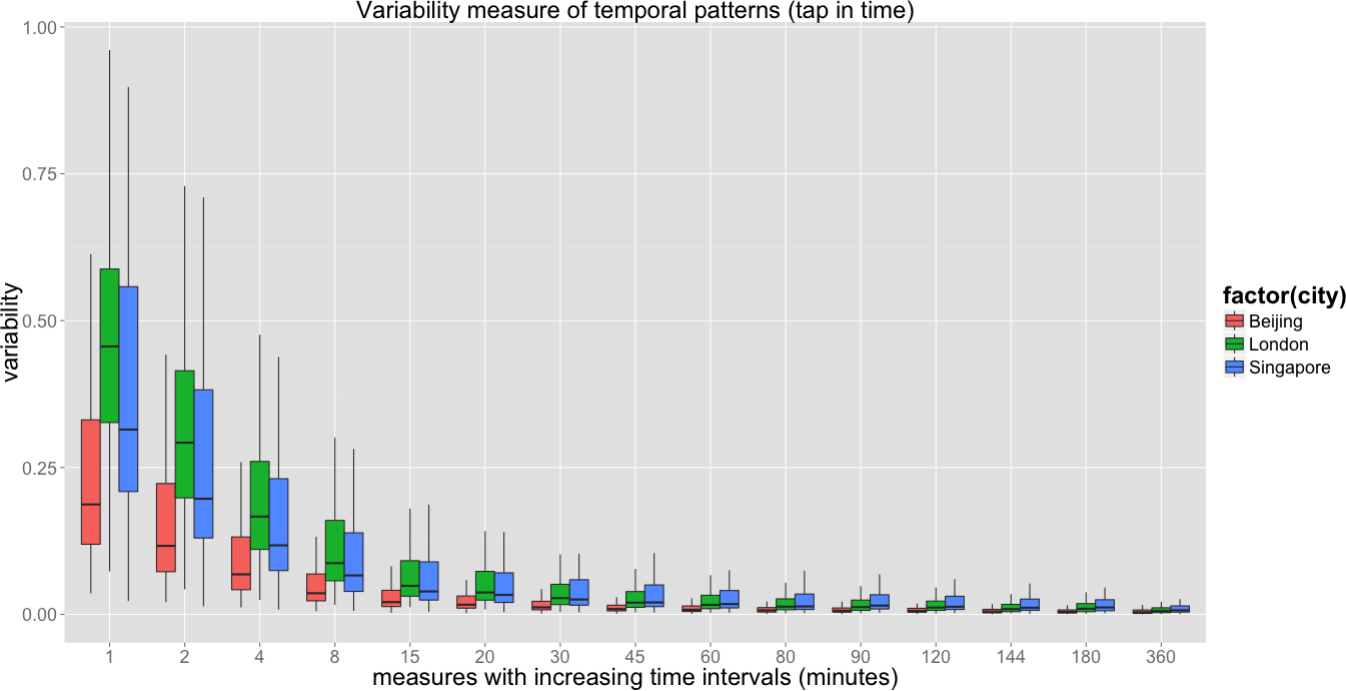
Comparative analysis of variability in temporal patterns.

We directly compare the three cities in this section, plotting the variability for these in Fig 6 The different sizes of metro system as well as the population of passengers have no influence on the variability measure, which is always normalized within [0,1] as is clear from Fig 6 that is another form of Fig 2. This shows that the Beijing metro system, although it moves the largest number of passengers and has the largest average flow per metro station, has the smallest variability across all measures. This means the flow volume in the Beijing underground is comparatively more predictable than others with Singapore in second place while it is clear that in London, this kind of travel is more difficult to predict.

It is possible that the complexity of the metro system might generate higher variability. From casual evidence of knowing the three systems, metro traffic on the London underground is quite often influenced by disruption such as signal failures and strike days. This could partially explain the larger variability of temporal patterns in that passengers stagger their journeys more than in other systems. The local demographics of passenger types could be another reason for all those who are 60+ years who reside in London can receive a free pass and this adds to greater variability particularly outside the peak hours. Moreover, these three cities are global cities which have large number of visitors and tourists contributing to a larger number of irregular, non-routine trips.

**Fig 7:**
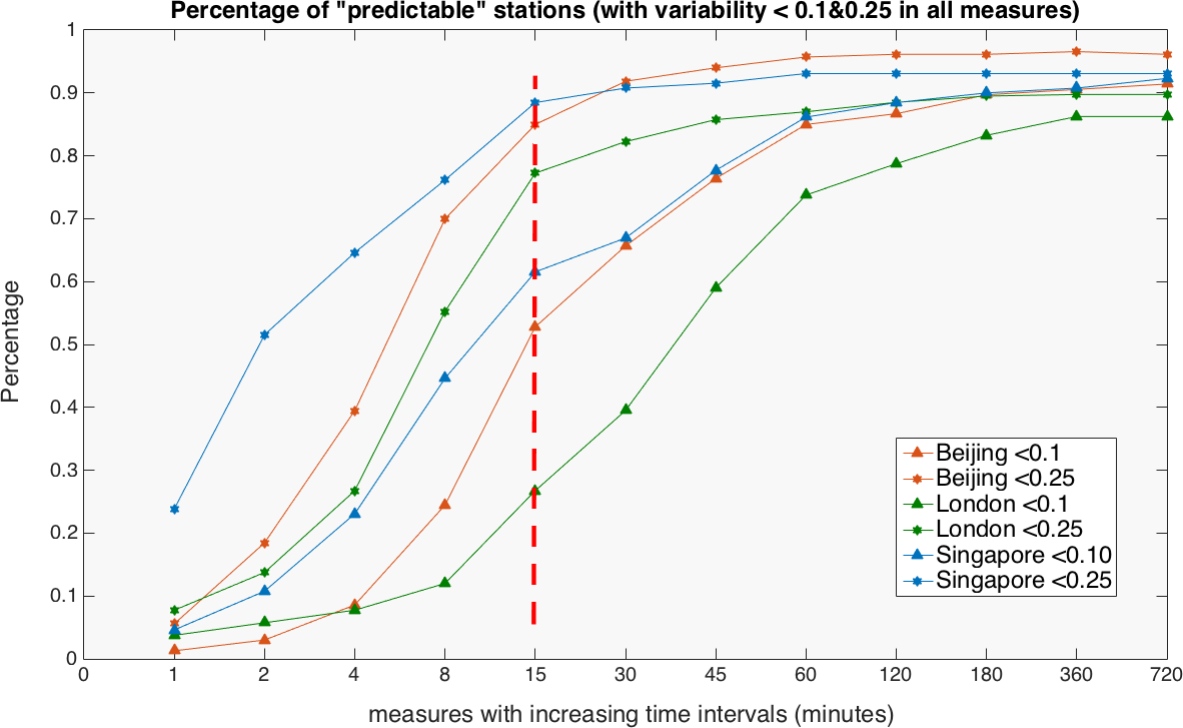
Comparative analysis of predictable trips origin and destinations. Note: A critical point exists at 15 minutes in all three cities as a universal pattern, which implies how closely we can predict future event.

In terms of origins and destinations of trips, we plot the percentage of ‘predictable’ stations in Fig 7. These are stations with variability smaller than a value *CVar*_*cutoff*_ at any measured time slot of a day. We then plot the percentage of predictable stations (*N*_*predictable*_ / *N*_*total*_) with *CVar*_*cutoff*_ = 0.1 and *CVar*_*cutoff*_ = 0.25. The percentage increases as the time intervals are aggregated. When the overall variability decreases along with increasing temporal scale as mentioned earlier, more stations fulfil the condition *CVar*_*cutoff*_ < 0.1 and *CVar*_*cutoff*_ < 0.25.

Although our goal is to characterize different cities and to determine if a universal pattern is found using these two sets of *CVar*_*cutoff*_ values, a critical point exists at 15 minutes, which implies how closely we can predict future events. Because the variability of the trip matrix increases sharply as the time slot decreases to less than 15 minutes, it is impossible to estimate where passengers travel unless further information on individual identification is considered even though real-time boarding information is captured at fare gates. It is a coincidence that the critical time slot obtained here is the same as the generally applied time interval for calculating the peak hour factor (PHF) originally proposed in highway engineering [34]. PHF is an indicator of the irregularity of peak hour demand, normally calculated as 4 times the 15 min peak in peak demand divided by the peak hour demand. This factor is important because transport infrastructure is usually designed using peak hour demand and its variability. As the headways of new heavy rail systems decrease to less than 2 minutes, it is reasonable to scale down the time interval to address short-term irregularity. Our results demonstrate that it is rather difficult to extract a stable PHF at a higher time resolution than 15 minutes.

**Fig 8:**
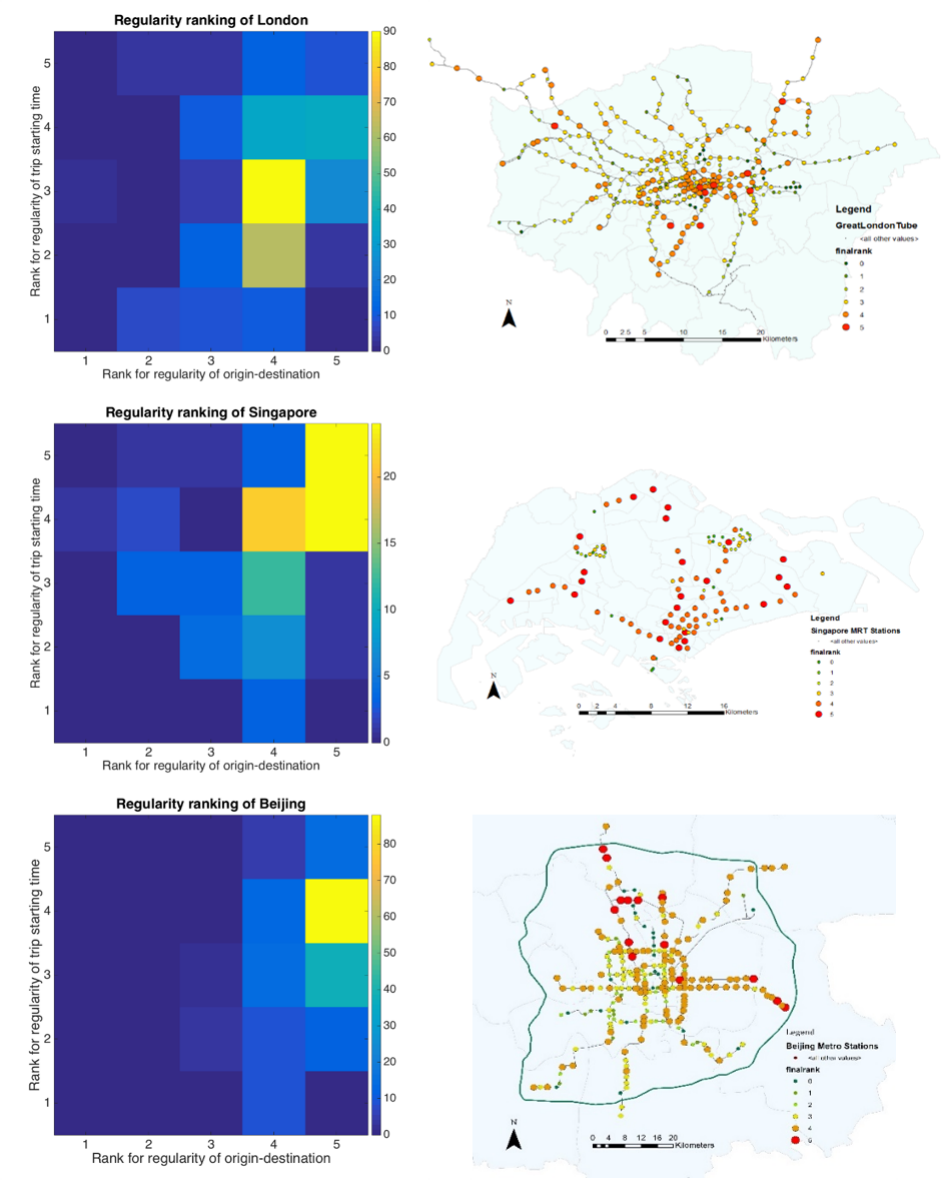
Combined ranking of when and where people travel. Note: Stations are ranked by *CVar*_*cutoff*_ = 0.1 and measured using temporal scales = 4, 15, 30, 60 and 180 (minutes). Stations which fulfill conditions at 4 minute temporal scales get the highest rank as 5 and so on. The geographic mapping is color coded by a combined score.

We further extend the way we define predictable stations using a ranking mechanism. Stations become more predicable in terms of trip flows at higher temporal resolutions and these get a higher ranking. Both temporal patterns of trip starting times *R*_*starting–time*_ and trip location patterns *R*_*O–D*_ are ranked at five levels as shown in Fig 8 (left). A combined rank is calculated as *R*_*combined*_ = min{*R*_*starting–time*_,*R*_*O–D*_} and mapped in Fig 8 (right). Both the statistical and geographical distributions of predictable stations are shown.

When we compare the three cities, we find that London is the most unpredictable of the three in terms of time, origin and destination of trips. The very dense distribution of metro station in the central area could well be the reason for many people have more than one choice of station for their journey and often this does not make a significant difference to their travel time. We are able to extract from the Oyster card data that many people take alternative routes, in particular for home-work trips and vice versa. Passenger management measures could well be another reason. For example, closing some of the fare gates at Bank and Camden Town, temporally holding passengers back from entering the station at Finsbury Park, closure of Victoria and Oxford Circus due to congestion at certain periods are frequently cited events that pertain to limits on capacity. These measures definitely increase the degree of irregularity in trip patterns

On the contrary, Singapore is the most predictable city in that its relatively simple and newer tube network is seldom affected by accident or train and signalling disruptions, and its planned polycentric urban form appears to produce a more smoothly flowing system than London. Sub-centres are built along the metro stations and surrounded by residential locations. Therefore, the choice of metro stations from which to travel is mostly influenced by relative distance and not so many alternative directions of travel on different lines are possible.

Beijing however has the highest regularity with respect to its temporal distribution of trips and is second with respect to station location. A number of reasons contribute to this. The most important one is the regular passenger control measure which is applied to about 40 stations where passengers are held outside the stations before being allowed to enter at regular time intervals during the morning peak. Such queues can last for miles. Passengers can either wait there, search and use an alternative station or mode according to their situation. This measure increases the temporal regularity by interfering with the randomness of arrival times and it decreases spatial regularity by forcing people to change their boarding stations randomly. Moreover, this inconsistency of temporal and spatial regularity can also ascribed to another unique phenomena in Beijing which is due to Vehicles Plate Number Traffic Restriction Measures (note: The restriction is based on the last digit on the license plate. For example, vehicles with a last plate digit of 2 or 7 must be off the road on Monday. Details of the restriction can be found in a bulletin posted on the website of the Beijing Traffic Management Bureau http://www.bjjtgl.gov.cn/) where many private car owners drive a car on most days but for one day use public transport system.

## Discussion and Conclusion

Here we have proposed a simple variability measure based on computing simple variances between any two profiles for detecting regularities and of metro trips associated with station and times of travel for three of the largest metro systems in world cities. This measure can be further used as part of an analytical framework for comparing cities, and for assessing the quality of data sets. In the case studies, we compared urban mobility patterns in London, Singapore and Beijing using one-week of smart card data, and although we would have preferred to have a longer time series, we are confident that these data provide a robust first analysis of the problem of variability. Two critical aspects of mobility patterns are examined, namely temporal distributions of trip starting times at stations and the pattern of trips that flow from and into given stations at different times taken from the O-D matrix. The diversity that this data captures shows important differences between cities with temporal patterns being quite stable in Beijing, notwithstanding special transport policies that do tend to interfere with the smooth running of the system. Singapore shows comparatively higher regularity in both temporal and location distributions which we consider due to better infrastructure and the configuration of its urban structure. London is the most complex of our three cities and this appears to be due to changes in travel behaviour from anticipated disruptions as well as from a high density of stations in its core.

Although this work focuses on variability in regularity, we have also found regularity in variability. That is, variability that also exists within universal patterns. In all cities, variability of temporal patterns increases with increased temporal resolution following a negative exponential function rather than a random distribution. The most predictable time period in all our analysis is in fact during the peak hours. The degree of regularity decreases dramatically from the 15-minute time interval, which implies that comparatively accurate predictions cannot be made for anything shorter than 15 minutes. In the same way we have analysed the temporal dimension, we show that a few stations are more predictable than the others. The density distribution of variability always follows a lognormal-like form for all measures and in summary, these findings imply that variability in regularity can be captured by the error variable which varies according to different temporal scales.

Much more remains to be researched following the concepts introduced here which are only preliminary. First, we need to further investigate variability in regularity across other dimensions, in particular, across spatial scales, and across different aggregations of individual behaviour to groups which are important for understanding urban dynamics. Furthermore, using variability in regularity of urban mobility to characterize many more cities through comparative studies is another direction which needs to be followed and we need to extend metro data to bus and related public transport data as well of course eventually to all modes. Smart-card data is useful for representing urban mobility at a fine granularity. But alternative data sets such as mobile phone data need to be explored in the same manner, In terms of factors exerting impacts on the different levels of variability, a more comprehensive urban analysis is needed. Finally, our motivation for this work originates from questions that relate to the accuracy of model simulations, which assume regularities often without exploring them in the manner we have introduced here. Our next effort is to incorporate the changes in variability into urban models for more accurate simulations of urban mobility.
